# Supplementary material for: Research on the coupling relationship and interaction between urbanization and eco-environment in urban agglomerations: A case study of the Chengdu-Chongqing urban agglomeration
Source: PLoS One. 2024 Mar 28;19(3):e0301287. doi: 10.1371/journal.pone.0301287 (PMC10977893; doi:10.1371/journal.pone.0301287)
Supplement: Attachment — Submitted filename: Review report PONE-D-23-26168_.docx [file pone.0301287.s003.docx]

The manuscript entitled “Research on the coupling relationship and interaction between

urbanization and environment in city clusters: A case study of the Chengdu-Chongqing

City Cluste”has been evaluated. The paper explores the dynamic evolution, coupling relationships, coordinated development patterns, and interaction mechanisms between urbanization and the environment by using Coupling Coordination Degree and Vector Autoregression models. I recommend accepting the manuscript directly if the following issues are addressed. The detailed comments are as below.

1. The innovation of the study is not clear. Please add this part.

2. There are minor errors in the introduction of formula variables in the article, such as line 186. Please check the whole text and make corrections.

3. In line 200, the range and significance of D can be indicated.

4. In line 218, the advantages of the VAR model and why it is suitable for the study of this paper compared with other methods can be added.

5. There is a problem with the paragraph formatting in line 648, please revise it.
