# Supplementary material for: Research on the coupling relationship and interaction between urbanization and eco-environment in urban agglomerations: A case study of the Chengdu-Chongqing urban agglomeration
Source: PLoS One. 2024 Mar 28;19(3):e0301287. doi: 10.1371/journal.pone.0301287 (PMC10977893; doi:10.1371/journal.pone.0301287)
Supplement: Attachment — Submitted filename: Response to Reviewers.docx [file pone.0301287.s004.docx]

Research on the coupling relationship and interaction between urbanization and eco-environment in Urban agglomerations: A case study of the Chengdu-Chongqing Urban agglomeration

**Weilong Wu ^1,2^, Ying Huang ^1^, Yuzhou Zhang ^2^, Bo Zhou ^1✉^**

**Responses to the reviewers’ comments**

Based on the comments and suggestions provided by the three reviewers, we have made the following modifications:

First, the introduction of the paper has been rewritten, focusing on the relationship between urbanization and the ecological environment. It discusses the background, research progress, necessity, and innovation of this study. Second, the introduction of the research area has been strengthened, particularly describing the current situation and contradictions between urbanization and the ecological environment in the research area, emphasizing the necessity of the study. Third, the theoretical explanation of the coupling relationship and interaction between urbanization and the ecological environment has been added. Fourth, the relevant content of VAR model testing has been strengthened, including tests for smoothness and cointegration analysis, as well as Granger causality analysis. Fifth, the discussion of the research results has been optimized. Sixth, all drawings have been reorganized according to the standardized format. Seventh, the content of the discussion section of the paper has been revised and improved.

The modifications we made in the revised version are marked in blue font. The specific replies are as follows:

**Reviewer #1**

1、The literature review does not provide a theoretical basis for the proposed method. There are at least four concepts of urbanization in this paper. It was not clearly specified to which this study refers.
 The first is to elaborate on the necessity of the research methods adopted in this article in the introduction. The second is to add theoretical analysis on the coupling relationship and interaction between urbanization and ecological environment in the second part of the article. These contents can be found in lines 90-109 and 148-191.

2、Before conducting the empirical study, this paper did not carry out a theoretical analysis of the relationship between the environment and urbanization, and the path of interaction between them was not clear.
 The theoretical analysis of the relationship between urbanization and ecological environment was added in the second part of the article, and the interaction mechanism between the two was analyzed. This content can be found from line 148 to line 191.

3、The indicator system needs further improvement. For example, there is an overlap in the meaning of SU5 and SU6, which all represent healthcare; and it lacks of air quality indicators in subsystem of ecological status, etc.
 The medical and health conditions are important aspects of social urbanization. In order to comprehensively reflect the medical and health levels of various cities, this paper also selects indicators such as the number of medical technicians per 10,000 people and the number of beds in healthcare institutions. In addition, this paper constructs an ecological environment system based on the PSR framework, which largely reflects the comprehensive results of indicators such as air quality, ecological pressure, ecological conditions, and ecological responses. Therefore, air quality indicators were not independently selected.

Of course, the construction of indicator system is an important task in exploring the coupling relationship between urbanization and ecological environment. The opinions of the reviewers have given us new insights, so we have added discussions and explanations about the evaluation indicator system in the discussion section of the paper. This content can be found in lines 623-658.

4、Result presentation is poor, as the results described in the text repeat information from the figures. Do not repeat the results. Be more creative, connect the results, point to the key information, provide some possible explanations for why the results show what they show,… The results as such are not the most important part of the research as they suffer from uncertainties. Instead the most important aspect is data discussion, interpretation (in understand the whole picture). The paper needs to be stronger in providing this.
 For questions with poor result presentation, we have re-discussed most of the results, reducing the description of chart information and increasing the summary of relevant patterns and explanations of phenomena. These contents can be found in sections 4.1.1, 4.1.2, 4.2.1, 4.2.2, and 4.3.2.2.

5、Why authors chose 2001, 2008, 2014 and 2020 as typical year to analyze the spatial and temporal evolution patterns of urbanization and environment? Do these time points have a special meaning?

The time range studied in this article is from 2001 to 2020, with a time span of 20 years, which is evenly divided into four periods for analysis. Therefore, 2001, 2008, 2014, and 2020 are chosen as typical years to analyze the spatiotemporal evolution pattern of urbanization and ecological environment.

6、In section 3.1, authors need to provide a basis for grading urbanization and environmental levels.

The basis for the classification of urbanization and ecological environment index has been added. Please refer to page 347.

7、line 406-408, the specific data is needed to support the results of the smoothness test and cointegration analysis.

The relevant results of VAR model smoothness test and cointegration analysis have been added. These contents can be found in section 4.3.1.

8、In the coupled coordination analysis, the paper analyzed the level of coupled coordination from the perspective of the whole and individual cities, respectively, while in the impulse analysis, it only analyzed the impulse response between the subsystems of the whole urban agglomeration and lacked the analysis of the impulse response between the environment and urbanization of each city.
 It is a good idea to analyze the pulse response of each city in the urban agglomeration, which can accurately grasp the interaction between urbanization and the ecological environment in each city. However, this study covers a total of 36 cities in the research area, and conducting pulse response analysis for each city would result in a large amount of content (252 drawings), which may be difficult to present in a research paper. Therefore, this part of the content was not included in the article.

9、The figures of the paper needs further revision, such as figure 1 cannot reflect the location of city clusters in China, figure 2 and 3 need to change the location of the legend, compass, and scale.

The relevant drawings have been standardized. See Figure 2-4.

**Reviewer #2**

1. There are not enough innovations, and there are many related studies on the coupling and coordination and spatio-temporal analysis of urban agglomerations in the Chengdu-Chongqing area.
 Although there are many articles on the research of the coupling and coordination of urbanization and ecological environment in the Chengdu-Chongqing urban agglomeration, most of them focus on the studies of coupling coordination, spatiotemporal patterns, and influencing factors. There is relatively little research on the interactive pressures and positive interactions between urbanization and ecological environment, especially the lack of research on the interaction at the level of systematic constituent elements.

Of course, the reviewer’s comments have greatly inspired us and refined the innovative points of the article. We have rewritten the content of the research review, focusing on the relationship between urbanization and the ecological environment. We have provided detailed discussions on the background, research progress, necessity, and innovation of this study. Additionally, we have strengthened the introduction of the research area, particularly by providing a comprehensive description of the current situation and contradictions between urbanization and the ecological environment in the research area, emphasizing the necessity of the study. Furthermore, we have conducted a theoretical analysis on the coupling relationship and interaction between urbanization and the ecological environment. These contents can be found in Sections 1 and 2 of the paper (pp. 39-191).

2. The format of the figure is not standardized.
The relevant drawings have been standardized. See Figure 2-4.

3. The policy recommendations are vague and insufficient, with no substantive policy recommendations, and it is recommended to further supplement the detailed policy recommendations according to the quantitative regression results.
 The policy recommendations were rewritten based on the research findings. This section can be found in pages 692-726.

4. There is no Granger causality test in the VAR model, which can be considered.
Added Granger causality test in VAR model. See content in 325-329, 4.3.1 (503).

5. The innovation points of this paper are not prominent, and it is recommended to clarify them at the end of the literature review.
 Added relevant discussions, see pages 90-109, 142-147.

6. The introduction part mentions the socio-ecological phenomenon of rapid urbanization, the relationship between urbanization and the environment, but the importance of coupling and interaction between them is insufficient, and it is suggested to add this part.

We have rewritten the content of the research review, focusing on the relationship between urbanization and the ecological environment. The background, progress, necessity, and innovation of the research are discussed in this article. At the same time, the introduction of the research area has been strengthened, particularly with regard to the current status and contradictions between urbanization and the ecological environment in the research area, providing a detailed description to emphasize the necessity of the research. See Introduction in Section 1 for more details.

**Reviewer #3**

1. The innovation of the study is not clear. Please add this part.

We have rewritten the content of the research review, focusing on the relationship between urbanization and the ecological environment. We have provided a detailed discussion on the background, research progress, necessity, and novelty of this study. The innovation points of the paper are summarized at the end of the research review. These contents can be found in lines 90-109 and 142-147.

2. There are minor errors in the introduction of formula variables in the article, such as line 186. Please check the whole text and make corrections.

The errors have been corrected and the entire text has been reviewed for corrections. See line 274.

3. In line 200, the range and significance of D can be indicated.

We have made the modifications, see lines 286-288.

4. In line 218, the advantages of the VAR model and why it is suitable for the study of this paper compared with other methods can be added.

According to the progress of the research and the needs of this study, this part of the content has been added to the literature review. See lines 90-109 for details.

5. There is a problem with the paragraph formatting in line 648, please revise it.

We have made the modifications, see lines 771.
